# Management of peritoneal surface metastases from colorectal cancer: Cytoreductive surgery, hyperthermic intraperitoneal chemotherapy, pressurized intraperitoneal chemotherapy, and beyond

**DOI:** 10.3389/fonc.2022.992030

**Published:** 2022-11-08

**Authors:** Christopher W. Mangieri, Edward A. Levine

**Affiliations:** ^1^ Department of Surgery, Tripler Army Medical Center, Honolulu, HI, United States; ^2^ Division of Surgical Oncology, Wake Forest Baptist Health Medical Center, Winston-Salem, NC, United States

**Keywords:** colorectal cancer, peritoneal surface malignancies (PSM), cytoreductive surgery and HIPEC, PIPAC, surgical standard

## Abstract

This article provides a contemporary review of the current surgical management of peritoneal surface malignancy (PSM) of colorectal origin. A brief review of the founding history of surgical intervention for PSM is followed by a focused review of the level I evidence, current clinical questions, and evolving advancements. While not intended to address all the facets of PSM, this review aims to provide the reader with the essential knowledge and resources to effectively provide surgical care for carcinomatosis due to colorectal malignancies.

## Introduction

The management of peritoneal surface malignancy (PSM) has significantly changed in both clinical attention and complexity over the past decade. PSM encompasses a broad range of etiologies to include rare primary peritoneal malignancies as well as the more commonly encountered secondary peritoneal metastatic disease. This is primarily due to the pioneering efforts establishing cytoreductive surgery with hyperthermic intraperitoneal chemotherapy (CRS-HIPEC) as an accepted therapeutic intervention within the oncologic community. While CRS-HIPEC constitutes the focus of the majority of research regarding surgical management of PSM, additional modalities such as adjuvant HIPEC and pressurized intraperitoneal aerosol chemotherapy (PIPAC) have also been investigated.

The heterogenicity of PSM, coupled with limited level I evidence, has made definition of the optimal management of PSM a difficult standard to define. The objective of this review is to provide a concise review regarding the history, current clinical research data, and future targets for surgical management of PSM, with the targeted audience being physicians who manage PSM to provide them with current standards within this evolving clinical field. The focus of this manuscript is for PSM of gastrointestinal origin, specifically colorectal and appendiceal primaries. Using the PICO (patient, intervention, comparison, outcomes) format, this literature review aims to educate the reader on current standard of care for patients with PSM secondary to appendiceal and colorectal primaries (P), undergoing CRS-HIPEC (I), evaluating the only published level I data (C), and establishing the standards of care (O).

## Subsections

### The origin of CRS-HIPEC

CRS-HIPEC has become the cornerstone for surgical management of PSM. CRS-HIPEC is currently the most utilized surgical intervention for PSM. Therefore, the overwhelming majority of basic science and clinical research data reside within the scope of CRS-HIPEC. The treatment tenet of CRS-HIPEC is two-pronged: the cornerstone being the removal of all gross tumor (CRS), and then utilizing “local” intraperitoneal adjuvant therapy, such as HIPEC, to effectively treat residual microscopic disease.

The genesis of CRS can be traced back to the 1930s with Dr. Meigs’ publication on his experience with cytoreduction for ovarian cancer (1). Further work in the 1960s–1970s by Dr. Griffiths’ National Cancer Institute group, investigating ovarian cancer, and Dr. Long’s Alabama group, investigating mucinous neoplasms, provided the first modern scientific evidence that CRS significantly increased survival for patients with PSM (2, 3). Both the research by Dr. Griffiths’ and Dr. Long’s collaboratives found that the addition of adjuvant systemic therapy improved outcomes (4, 5). The conceptual basis of CRS is quite elementary; if all gross diseases can be resected, than there will be significant benefit. Those inceptive efforts with CRS provided the “proof of concept” work facilitating the evolution of surgical management of PSM.

The practice of combined CRS-HIPEC was spearheaded by Dr. Paul Sugarbaker’s experience with the Washington Cancer Institute in the 1980s. That pioneering work combining CRS with HIPEC established the conduct of CRS-HIPEC as it is utilized currently. Progressing the landmark work by Dr. Spratt and colleagues from the University of Louisville, who developed a human delivery system for HIPEC, Dr. Sugarbaker’s group became the lead academic research team evaluating CRS-HIPEC providing critical prospective data to legitimize CRS-HIPEC (6–9). Several benefits of HIPEC over adjuvant systemic therapy have been proposed. HIPEC has been touted as having a superior therapeutic effect within the peritoneal cavity where systemic therapy penetration is limited (10). Further, intracavitary administration of chemotherapy is able to achieve higher, more tumoricidal, levels of chemotherapy at the site of the disease than can be accomplished with even the most aggressive dosing of systemic drug can achieve without undue toxicity (10). Lastly, the one-time dosing of HIPEC is more cost effective (compared with repeated systemic chemotherapy dosing) and is not associated with the compliance issues or tolerability of multicycle therapy (10). That collective early CRS-HIPEC endeavor by the multiple founding PSM surgeons has served as the standard for surgical management of peritoneal carcinomatosis for nearly 40 years. Only recently with the results from the PRODIGE7 trial has the decoupling of CRS and HIPEC been seriously reconsidered.

The ability to efficiently and accurately quantify the volume of peritoneal metastatic disease across multiple pathologies and applying that metric to clinical decision making was also established by Sugarbaker and his colleagues. They developed the peritoneal cancer index (PCI) score which allowed PSM surgeons to reliably quantify burden of disease on a point spectrum from 1 to 39 based on standardized anatomical locations (11, 12). The PCI is the standard currently utilized at most larger centers for quantification of the amount of disease present at the start of CRS.

The completeness of the cytoreduction (CC) score was invented by Dr. Glehen and Dr. Gilly to grade the quality of CRS (13). Slight technical variations of that CC score, R score (from the AJCC staging manual), are used at differing PSM centers, but the primary gradation scheme is similar, with a complete cytoreduction (CC-0, R1) defined as no gross residual tumor, a near-complete cytoreduction (CC-1, R2a) with less than 2.5 mm of gross residual disease remaining, and two incomplete cytoreduction scores for residual disease between 2.5 mm and 2.5 cm (CC-2, R2b) and residual disease in excess of 2.5 cm (CC-3, R2c). Similar to how CRS-HIPEC is a bimodal intervention, the PCI-CC score provides two complimentary data points. The PCI score provides guidance for PSM surgeons regarding the likelihood of achieving a complete cytoreduction based on the underlying tumor biology, while the CC score has become the main determining factor to proceed with HIPEC at the time of CRS. The collection of promising initial phase I and II trials evaluating CRS-HIPEC paired with the reproducible PCI-CC scoring scheme provided the requisite groundwork for the subsequent phase III studies validating surgical management of PSM.

### CRS as standard oncologic therapy for PSM for colorectal and appendiceal primaries and utility of HIPEC in flux

CRS-HIPEC has been frequently performed at leading academic centers worldwide since the 1990s, but it took nearly two decades for the oncologic community to embrace surgical management of PSM as standard of care. Despite positive basic science and clinical data with CRS-HIPEC, surgical management of PSM was often labeled radical therapy leading to limited utilization. The inertia to CRS-HIPEC was multifaceted. One of the major hurdles was the general therapeutic nihilism with treating diffusely metastatic diseases. Despite objective effectiveness with CRS-HIPEC, that evidence was frequently trivialized within the oncologic academic oncologic community (14, 15). The general negative bias toward CRS-HIPEC was further reinforced by the initial morbidity and mortality associated with the procedure that was in the 60% and 20% range, respectively (16). Like with most initially labeled radical therapies, persistent hesitance is only overcome with establishing gold standard, phase III, trial data, despite the fact that the median survival with PSM from GI primaries prior to the turn of the century was a mere 6 months.

To date, there have only been four completed and reported randomized control trials (RCTs) evaluating the primary surgical management of PSM for colorectal cancer, and one for appendiceal. All of those trials involved secondary metastatic disease to the peritoneal cavity, of which three studies focused on colorectal and appendiceal primaries with the remaining study evaluating carcinomatosis due to ovarian cancer. As will be detailed in the succeeding text, those RCTs, particularly the trials involving colorectal primaries, have definitively established CRS as standard-of-care oncologic management for patients with carcinomatosis. However, the most recently published trial, PRODIGE7, has now reintroduced additional discussions on the surgical management of PSM. Does HIPEC after CRS provide any additional benefit? The level I data for CRS-HIPEC for PSM secondary to gastrointestinal cancer, colorectal, and appendiceal tumors will be evaluated in detail, specifically examining each published individual RCT followed by a discussion of the current quandary regarding the benefit of HIPEC.

### The Dutch trial

The first RCT was by Verwaal et al., from the Netherlands Cancer Institute, published in 2003 (17). In that trial, 105 patients with peritoneal carcinomatosis due to colorectal primary tumors, of which approximately 85% were colorectal cancer and 15% were appendiceal cancer, were equally randomized to a control standard chemotherapy-alone group and an experimental CRS-HIPEC group. The control group received 5-FU and leucovorin alone (standard systemic chemotherapy at the time the trial was started). Patients randomized to CRS-HIPEC were to undergo an optimal cytoreduction, defined for this study protocol as R2a or better, with mitomycin C as the HIPEC agent.

The median follow-up was 21.6 months, and the primary outcome was survival. For the CRS-HIPEC cohort, an R1 resection was achieved in 33%, R2a in 39%, and R2b in 18% with a surgical mortality rate of 8%. The CRS-HIPEC group had significantly improved survival with a median OS of 22.4 vs. 12.6 months (HR = 0.55, 95% CI 0.32–0.95, P = 0.032). Subgroup analysis based on sex, age, tumor site, and either primary or recurrent disease revealed no heterozygosity from the main results, and CRS-HIPEC was significantly beneficial across all subgroups. Further evaluation of CRS-HIPEC stratified by burden of disease and completeness of cytoreduction, R1/R2a vs. R2b, demonstrated improved survival with decreased disease burden and optima cytoreduction with median OS of 29 vs. 5.4 months (P < 0.0001) and 20 vs. 5 months (P < 0.0001), respectively. Of note, PCI scoring was not utilized in this trial. Burden of disease was quantified by abdominal regions, ≤5 or 6–7, containing disease. Long-term trial data analysis found that CRS-HIPEC had persistent utility with improved DFS and PFS of 22.2 vs. 12.6 months (P = 0.028) and 12.6 vs. 7.7 months (P = 0.020), respectively (18). Also for those achieving an R1 cytoreduction, the 5-year OS rate was 45% on that 8-year follow-up which was remarkably improved survival compared to historical data with standard chemotherapy alone.

The Dutch trial was a true landmark study in several regards. It provided the first level I evidence evaluating CRS-HIPEC and most importantly produced favorable data demonstrating improved survival with CRS-HIPEC. However, critics of the trial suggested that the improved survival with CRS-HIPEC compared to the control group was due to utilization of an outdated less efficacious chemotherapy regimen. At the time of the publication of the trial, newer oxaliplatin and irinotecan-based chemotherapies FOLFOX and FOLFIRI were becoming the standard of care for systemic treatment of metastatic colorectal cancer based on their superior results (19). Despite those study critiques, the Dutch trial was instrumental in validating CRS-HIPEC as both a therapeutic intervention with true utility and as a surgery that could be safely performed.

### The Swedish trial

The next RCT was performed by Cashin et al., as a study involving several academic centers within Sweden (20). Unfortunately, the trial was terminated early due to poor accrual with only 48 patients recruited at the time of study closure. Publication of the trial data occurred in 2016. All the study participants had secondary peritoneal carcinomatosis with a near-identical distribution of primary etiology to the Dutch trial, approximately 85% being colorectal tumors and 15% being appendiceal tumors. Each study group contained 24 patients who were equally randomized. The control group received contemporary standard systemic chemotherapy with FOLFOX. The experimental CRS-HIPEC group had the same objective as the Dutch trial with trying to achieve an optimal cytoreduction of CC-0 or CC-1. However, the HIPEC protocol in the Swedish trial was performed in an adjuvant technique utilizing an abdominal port to infuse 5-FU with leucovorin every 4–5 weeks for a total of 6 cycles.

The median follow-up was 78 months with survival as the primary outcome. For the CRS-HIPEC group, the mean PCI was 18 and 79% of cases achieved either a CC-0 or CC-1 cytoreduction. The surgical mortality and morbidity, defined as Clavien–Dindo III/IV complications, rates were 0% and 33%, respectively. For the entire study population, the CRS-HIPEC group had superior survival with a median OS of 25 vs. 18 months and 2-year OS rates of 54% vs. 38% (HR = 0.51, 95% CI 0.27–0.96, P = 0.04). Yet, for the entire study population on multivariate analysis, CRS-HIPEC was not independently associated with improved OS (HR = 2.17, 95% CI 0.77–1.61, P = 0.14). Adjusting only for patients who achieved an optimal cytoreduction, CC-0 or CC-1, CRS-HIPEC significantly improved OS on both univariate (HR = 0.20, 95% CI 0.09–0.45, P = 0.0001) and multivariate (HR = 0.11, 95% CI 0.04–0.34, P = 0.0005) analyses. The median OS was 40 months with a 5-year OS rate of 40% in those achieving an optimal cytoreduction. There was no significant difference in PFS with a median PFS of 12 vs. 11 months, but based on 5-year PFS rates, there was a trend toward improved outcomes with CRS-HIPEC at 17% vs. 0% (P = 0.16).

The Swedish trial justifiably received less acclaim than the Dutch trial due to the fact that it failed to accrue the required number of participants to satisfy its study design. Despite the early termination, this incomplete study still provided objective data that further supported CRS-HIPEC. Again, a statistically significant survival benefit was demonstrated with CRS-HIPEC in the Swedish trial. Those findings strengthened the results from the Dutch trial since the control group participants in the Swedish trial received the contemporary standard of FOLFOX systemic therapy. Therefore, the criticism of improved survival with CRS-HIPEC levied in the Dutch trial due to the use of non-contemporary chemotherapy regimens was mitigated with the Swedish trial results. Evaluating the Swedish trial with the perspective of the PRODIGE7, PROPHYLOCHIP, and COLOPEC trial data, which will be subsequently discussed, these study results suggest that CRS alone is the principal factor in improved survival with surgical management of PSM.

### The French PRODIGE7 trial

The most recent RCT evaluating CRS-HIPEC for colorectal cancer was PRODIGE7 performed by Quénet et al. and published in 2021 and is arguably the most influential trial regarding surgical management of PSM (21). PRODIGE7 is the largest trial being a multicenter study, involving French PSM institutions, of 265 patients with peritoneal carcinomatosis all due to colorectal adenocarcinoma alone. There were no appendiceal cancer cases allowed in PRODIGE7, which differentiated it from the previously performed Dutch and Swedish trials. Another critical distinguishing feature of PRODIGE7 was it equally randomized patients to a CRS-alone group and a CRS-HIPEC group. The final study population consisted of 133 patients in the CRS-alone group and 132 patients in the CRS-HIPEC group. All study participants had to have a PCI ≤25 and undergo an optimal cytoreduction defined as no gross residual disease or remaining tumor implants of ≤1 mm, modified CC-1/R2a cytoreduction, in order to be included in the final analysis. The study HIPEC protocol significantly differed from many other PSM centers with a shortened 30-min perfusion of oxaliplatin that was combined with an IV dose of 5-FU. Lastly, nearly the entire study populace, over 95% received systemic therapy either preoperatively or as a postoperative adjuvant, with approximately 65% of patients receiving both preoperative and postoperative adjuvant systemic chemotherapies.

The median follow-up was 63.8 months, and the primary endpoint was OS with RFS as a secondary outcome. Both cohorts had excellent cytoreductions with approximately 90% of all patients achieving an CC-0/R1 cytoreduction with the remaining 10% undergoing a modified optimal cytoreduction. The median PCI scores were 9 and 10 for CRS and CRS-HIPEC, respectively. There was no difference in survival outcomes between the cohorts. The median OS was 41.2 vs. 41.7 months and 5-year OS rates of 36.7% and 39.4% (HR = 1.0, 95% CI 0.63–1.58, P = 0.99) for CRS alone and CRS-HIPEC, respectively. Likewise, there was no difference in RFS with median RFS of 11.1 vs. 13.1 months and 5-year RFS rates of 13.1% vs. 14.8% (HR = 0.91, 95% CI 0.71–1.15, P = 0.43) for CRS and CRS-HIPEC, respectively. On subgroup analysis to include sex, primary location, nodal status, neoadjuvant versus adjuvant chemotherapy, and completeness of cytoreduction, there was no heterozygosity with the main analysis. The only subgroup that benefited from CRS-HIPEC, for OS alone, was for cases with an intermediate PCI score of 11-15 (HR = 0.44, 95% CI 0.21–0.99). There was no significant difference in mortality rates between CRS and CRS-HIPEC, 4.5% vs. 6%, as well as no difference in 30-day complication rates at 32% vs. 42% (P = 0.083). However, CRS-HIPEC was associated with an increased long-term—31–60 days—complication rate at 26% vs. 15% (P = 0.035).

To date, the survival results from PRODIGE7 are the best for any prospective study evaluating metastatic colorectal cancer regardless of intervention. However, the results of PRODIGE7 have become a flashpoint subject within the academic oncology community. Generally speaking, the medical oncology community has viewed the study results as a negative trial since there was no overall survival benefit with HIPEC. However, within the surgical oncology viewpoint of the study, it is more appropriately evaluated as the trial that has clearly established CRS as the optimal oncologic therapy for appropriate candidates. As previously mentioned, the median OS times within PRODIGE7 have not been rivaled by any other therapeutic intervention. While there was no difference in survival between CRS alone and CRS-HIPEC, all patients underwent cytoreduction; therefore, it is the CRS that improves survival. That finding is strengthened by the fact that nearly all patients received systemic therapy. Further, the OS benefit seen in the intermediate PCI range of 11–15 suggests an effect of the HIPEC. Hence, it is the combination of systemic therapy and CRS that produces the best survival for patients with metastatic colorectal cancer limited to the peritoneal cavity. While HIPEC was not found to add any benefit in the entire population of PRODIGE7 patients, the specific HIPEC study protocol has been strongly criticized for being an outlier from many leading PSM centers (22). Due to that concern with the HIPEC protocol, the French Cancer Consortium (PRODIGE) is currently establishing another RCT to replicate the PRODIGE7 trial with a HIPEC protocol that is more consistent with PSM center norms, particularly longer HIPEC treatment time. Despite the legitimate concerns about PRODIGE7 questioning the utility of HIPEC, the consensus of expert PSM surgeons is that PRODIGE7 has unequivocally established CRS, combined with systemic therapy, as the standard of care for carcinomatosis of colorectal origin for appropriate CRS candidates.

### The appendiceal randomized trial

To date, there has been a single completed and reported prospective randomized trial for PSM from appendiceal sources. That study by Levine et al. was a prospective randomized trial evaluating the utility of mitomycin vs. oxaliplatin in the HIPEC perfusate after CRS (23). The study was performed at Wake Forest University, M.D. Anderson, and the University of Pittsburgh Medical Center. Principal endpoints were survival, quality of life (24) and hematologic toxicity of the two agents. Patients with mucinous appendiceal neoplasms with evidence of peritoneal dissemination were consented and underwent cytoreductive surgery and HIPEC using a closed technique for 120 min. Patients were randomized intraoperatively to HIPEC using mitomycin (40 mg) or oxaliplatin (200 mg/M^2^). Follow-up included daily blood counts and toxicity assessments using CTCAE criteria (volume 3.0) and quality-of-life measures.

A total of 121 analytic patients were accrued to the trial over 6 years at three sites. The cases were 57% women, with an average age of 55.3 years (range 22–82). The disease was low grade in 77% and high grade in 23%. There were no significant differences in hemoglobin or platelet counts. The WBC was significantly lower in the mitomycin group between postoperative days 5–10. Quality-of-life scores were better in the oxaliplatin group for physical wellbeing (24.2 vs. 22.4, p = .015) and emotional wellbeing (19.4 vs. 18.0, p = .048) through 1 year after surgery. Overall survival and disease-free survival at 3 years were similar at 83.7% and 66.8% for mitomycin and 86.9% and 64.8% for oxaliplatin, respectively.

This study represents the first completed prospective randomized trial of cancer of the appendix in any setting and shows that despite their rarity, multicenter trials for appendiceal neoplasms are feasible. Both mitomycin and oxaliplatin are associated with minor hematologic toxicity. However, mitomycin has slightly higher hematologic toxicity and lower QOL than oxaliplatin in HIPEC. The overall survival was similar with the two agents. This similar survival suggests either equal efficacy or the lack of efficacy for either agent. Consequently, if HIPEC is to be delivered after CRS for appendiceal PSM, oxaliplatin may be preferred in patients with leukopenia and mitomycin preferred in patients with thrombocytopenia due to prior chemotherapy. However, based upon the superior quality-of-life data and lower cost, oxaliplatin is considered the default agent for HIPEC for appendiceal cancer by the authors (23, 24).

### The future of HIPEC

The PRODIGE7 results have returned clinical scrutiny to the efficacy of HIPEC for PSM due to colorectal tumors (21). Since the emergence of standardized surgical therapy for PSM, the pairing of CRS-HIPEC has been an unquestioned pairing with accepted synergy. The initial endeavors by the pioneer PSM surgeons to have therapeutic surgical management of carcinomatosis considered acceptable and efficacious required legitimization of CRS-HIPEC as dual therapy *via* prospective randomized trials. Now with the reverberations of the PRODIGE7 results, combined with the other level I evidence, there is no question about the utility of CRS. However, the current clinical conundrum is: should we continue with the longstanding approach of pairing CRS with HIPEC?

There are several reasons to temper the inclination to dismiss the role of HIPEC based on the PRODIGE7 results. First, as mentioned previously, there is sound critical concern of the HIPEC protocol utilized with PRODIGE7. The incredibly short perfusion time of 30 min, nearly all leading HIPEC centers perfusing for 1–2 h, and the choice of oxaliplatin as the perfusate are grounds enough to question the clinical applicability of PRODIGE7 when it comes to evaluating the role of HIPEC. While mitomycin C is the most commonly selected HIPEC agent, there are prospective randomized data that demonstrate no survival difference between mitomycin or oxaliplatin HIPEC (23), albeit for appendiceal and not colorectal cancer. With the planed performance of another PRODIGE RCT to better evaluate the true efficacy of HIPEC using a more accepted protocol, future study results will better delineate the future of HIPEC.

Another fundamental question when applying the PRODIGE7 results to the utility of HIPEC is: how does the completeness of cytoreduction influence the efficacy of HIPEC? There is a large volume of high-quality data corroborating a complete cytoreduction, CC-0/R1, as the most prognostic independent variable for survival (24–27). One of the true feats of the PRODIGE7 trial was that 90% of study participants underwent a CC-0/R1 cytoreduction, while subgroup analysis revealed no difference with the main results. Specifically, regardless of the completeness of cytoreduction, an outcome variable, an argument can be made that with so few CC-1/R2a cases in the PRODIGE7 population, a true determination of HIPEC efficacy with residual disease cannot be determined.

Despite years of research, there is an ongoing debate regarding the mechanism of effectiveness of HIPEC in clinical practice. Data on tissue penetration of HIPEC are acquired from animal studies where the maximum depth into the peritoneum was measured between 1 and 5 mm (28, 29). There are limited human data on the effective therapeutic penetration of HIPEC (30, 31). Thus, since the advent of CRS-HIPEC, a residual tumor goal of less than 2.5-mm implants, CC-1/R2a or better cytoreduction, has been the threshold to proceed with HIPEC with an expected therapeutic effect (32). We clearly have suboptimal understanding of the extent of efficacy with HIPEC. In fact, recent analysis has even found that HIPEC may hold a survival benefit for incomplete cytoreductions, CC-2/R2b and CC-3/R2c, for certain patient populations (33). HIPEC already has a well-established role as palliative treatment for management of malignant ascites (33–35).

Until there is more definitive level I evidence to adjudicate the utility of HIPEC in this setting, it is expected that most PSM centers will continue to perform CRS-HIPEC as opposed to CRS alone. Further, scientific evaluation of HIPEC itself is certainly required to ensure it has a meaningful benefit in the oncologic management of PSM. However, it should be stated that the authors feel that continuing to pair CRS with HIPEC is likely a favorable risk–benefit ratio. The individual HIPEC component is a limited factor in the associated morbidity of CRS-HIPEC compared to the major visceral resections and anastomoses involved with the actual cytoreduction (36, 37). Considering all of the knowns and unknowns with HIPEC, it seems prudent for the PSM surgeon to continue with the established coupling of CRS-HIPEC. However, preoperative discussions with patients of the risk and benefits of the HIPEC component of this treatment are in order.

### Adjuvant HIPEC, PIPAC, and future therapy

With the positive results from the Dutch and Swedish RCT data in conjunction with the additional literature on the benefit of HIPEC, there was keen academic interest to determine if adjuvant HIPEC had the potential to prevent carcinomatosis, particularly with the application of adjuvant HIPEC in those cases assessed at high risk for the subsequent development of PSM. There was sound basic science and clinical reasoning underlying the premise of adjuvant HIPEC, as well as promising initial research data (38, 39). However, the final results of two RCTs found that adjuvant HIPEC lacked any demonstrable efficacy.

The first adjuvant HIPEC RCT was COLOPEC. This trial was a multicenter study of 204 patients with resected colorectal cancer that were assessed as high-risk for peritoneal recurrence based on advanced stage disease (T4N0-2M0) or primary tumor perforation (40). Patients were equally randomized to a control group of structured surveillance or experimental adjuvant HIPEC group. There was no difference in survival outcomes between the cohorts. The 18-month peritoneal metastasis-free survival rates were 80.9% vs. 76.2% (P = 0.28) for HIPEC and surveillance, respectively. There was also no difference in 18-month DFS (69% vs. 69.3%, P = 0.99) and OS (93% vs. 94.1%, P = 0.82). The second published RCT was PROPHYLOCHIP, another multicenter trial involving 150 patients with resected high-risk colorectal cancer based on either a perforated primary tumor or a small-volume peritoneal disease at the index surgery that was completely resected (41). Again, patients were equally randomized to either a control structured surveillance group or an experimental second-look surgery adjuvant HIPEC group. After a median follow-up of over 50 months, there was no difference in the primary outcomes of 3-year DFS and OS rates. The negative results of both COLOPEC and PROPHYLOCHIP essentially quelled the performance of adjuvant HIPEC after resection of high-risk colorectal cancer. Those combined trial findings suggest that HIPEC is ineffective when no gross residual disease is present. Interestingly, if you apply the PROPHLYOCHIP and COLOPEC conclusions, HIPEC is ineffective when no gross residual disease remains, to the PRODIGE7 results that lack of utility with HIPEC in PRODIGE7 may be confounded by the fact that the majority of patients had no residual disease at the time of actual HIPEC. Although the COLOPEC and PROPHYLOCHIP trials found no benefit with adjuvant HIPEC, the initial results from the HIPECT4 RCT demonstrated that there was utility with adjuvant HIPEC for T4 colorectal tumors that underwent an oncologic resection (42).

The most recent advancement with surgical management of PSM is the development of pressurized intraperitoneal chemotherapy (PIPAC). The theoretical basis of PIPAC is the potential ability to deliver more efficacious intraperitoneal chemotherapy treatments repeatedly *via* an intraperitoneal nebulizer device. Proponents of PIPAC claim that approach superior to HIPEC *via* the ability of PIPAC to improve peritoneal distribution with enhanced tissue uptake while also being better tolerated than HIPEC with the ability for the procedure to be serially repeated in a minimally invasive fashion (43, 44). Additionally, PIPAC is touted as a therapeutic intervention for all patients with PSM, regardless of etiology or functional status, as opposed to HIPEC which is typically limited to patients with certain etiologies who are good surgical candidates for a major operative procedure. Currently, PIPAC is limited to palliative therapy with the rationale being that most patients with carcinomatosis will never be appropriate candidates for CRS-HIPEC and therefore will be limited to systemic therapy which has limited therapeutic effect on peritoneal-based metastatic disease. PIPAC allows direct delivery of the same chemotherapy agents to PSM which enhances effectiveness. A European collaborative group published the first basic science experience with PIPAC in an animal model in 2000, but it took that same group nearly a decade to develop a delivery system appropriate for human clinical use (45, 46). Currently, there is only one manufactured nebulizer device (CapnoPen^®^ Villingendorf, Germany) available for clinical application which must be delivered in a minimally invasive fashion utilizing CO^2^ insufflation. The clinical experience with PIPAC was principally in Europe. While the collective experience with PIPAC is significantly more limited compared to HIPEC, a recent systematic review, including over 1,800 cases, suggested that there was oncologic efficacy in 50%–80% of cases that were previously refractory to standard systemic therapy (47). PIPAC has definite potential to be a more encompassing therapeutic option for surgical management of PSM but it may be associated with increased complications compared to HIPEC (48). Prospective trials are ongoing to provide required high-level evidence to support more ubiquitous use of PIPAC. Although PIPAC is delivered *via* minimally invasive techniques, there is additional risk of aspiration of vaporized chemotherapy to operative teams and the agent is commonly delivered after all personnel leave the operative theater until the vapor is likely cleared.

A conundrum with intraperitoneal chemotherapy has always been, which is the optimal perfusate agent for a particular histology. Since coveted level I evidence is quite limited for intraperitoneal chemotherapy as a whole, PSM surgeons are rather handicapped in accurate prognostic therapeutic choices for individual patients. Due to the inherent difficulties in completing RCTs for PSM, there likely may never be adequate level I data to support the multitude of clinical decision points (47, 49). Therefore, non-standard approaches need to be utilized to pair the most efficacious HIPEC, or PIPAC, agent for the specific tumor characteristics of the patient. As modern oncologic care becomes exponentially more technically advanced, the treatment paradigm is shifting from “one-size fits all” to precision medicine. The use of organoids as a therapeutic treatment platform has the potential to completely change cancer care by providing real-time treatment data for how a patient’s unique tumor will respond to a plethora of agents (50). Validating the use of organoid-derived treatment data for clinical application in PSM has been published with promising results (51–54). With further “proof of concept” and clinical data, organoid platforms could become the critical tool to providing reliable treatment guidance that likely will never be obtainable with level I evidence for PSM.

### Consensus management standards

An Achilles’ heel regarding management of PSM has been the lack of recognized clinical practice guidelines by an accepted expert consortium. Varying factors have contributed to that dilemma: the lack of high-quality large-volume data to extrapolate guidelines from, the fact that PSM has been a relative “orphan” disease, and the wide spectrum of different etiologies that fall under the PSM umbrella. In 2020, the Chicago Consensus Working Group published a comprehensive set of multidisciplinary clinical practice guidelines for the management of PSM (54). This was a monumentous achievement that united preeminent experts throughout North America to provide the first set of universally well-accepted standards for PSM. The Chicago Consensus guidelines not only provided guidance for general standards in the multidisciplinary management of PSM but also provided etiology-specific recommendations for management of carcinomatosis secondary to appendiceal neoplasms, colorectal cancer, peritoneal mesothelioma, gastric cancer, ovarian neoplasm, neuroendocrine tumors, and rare primaries such as breast and GIST (55–62). In addition to recommendations for therapy with curative intent, the Chicago Consensus Working Group provided guidance for palliative management of PSM as well (63). Any physician who manages patients with PSM should be well versed in the Chicago Consensus guidelines as they are considered a current standard. While the field of PSM remains dynamic, it is anticipated that the Chicago Consensus Working Group will remain a central authority in compiling leading data and expertise to provide recommendations that are applicable to the entire spectrum of PSM centers.

## Conclusions

This review article provides physicians who manage PSM, primarily of colorectal origin, with the landmark trial data and current guiding standards. It is beyond the scope of this manuscript to comprehensively address every subset of PSM. The reader is strongly encouraged to examine the references. The authors recommend the Chicago Consensus Guidelines, for further guidance of the subject matter. While the full extent of PSM management cannot be adequately covered in a review article, the quintessential principles and resources have been discussed.

The field of therapy for PSM continues to expand and become a more commonly treated cancer. No longer is therapeutic nihilism appropriate when peritoneal metastases are encountered. The surgical management of PSM/carcinomatosis should no longer be considered a radical procedure. CRS has clearly been proven through level I evidence to provide a significant survival benefit for appropriate patients. Until there is definitive evidence that HIPEC is not beneficial, CRS-HIPEC will continue to be paired together to provide the optimal curative therapy for patients with PSM due to colorectal malignancies. Additionally, innovative advances like PIPAC and organoid platforms and genomics could provide the framework to expand surgical management of PSM to a larger patient pool. Lastly, what previously was a discipline of often non-networked experts, without clear field defining standards, now has a collective and well-recognized community, as the Chicago Consensus Working Group has shown.

## Author contributions

All authors listed have made a substantial, direct, and intellectual contribution to the work, and approved it for publication.

## Conflict of interest

The authors declare that the research was conducted in the absence of any commercial or financial relationships that could be construed as a potential conflict of interest.

## Publisher’s note

All claims expressed in this article are solely those of the authors and do not necessarily represent those of their affiliated organizations, or those of the publisher, the editors and the reviewers. Any product that may be evaluated in this article, or claim that may be made by its manufacturer, is not guaranteed or endorsed by the publisher.
